# Improvement of quality of life by conservative management of thoracic scoliosis at 172°: a case report

**DOI:** 10.25122/jml-2021-0332

**Published:** 2022-01

**Authors:** Eric Chun-Pu Chu

**Affiliations:** 1. New York Chiropractic and Physiotherapy Centre, Hong Kong, China

**Keywords:** chiropractic, retinoblastoma, thoracic scoliosis, spinal manipulation, DEXA – dual-energy x-ray absorptiometry, MRI – magnetic resonance imaging, NPS – numeric pain scale, NSAIDs – non-steroidal anti-inflammatory drugs, pRB – retinoblastoma protein, QoL – quality of life, RB – retinoblastoma gene, WHOQOL – World Health Organization Quality of Life

## Abstract

Adult scoliosis is a sideways curvature of the spine causing bilateral lower back pain and paresthesia of the lower limbs. Conservative treatment for scoliosis is primarily performed for youth, but scoliosis can be deteriorating as the patient ages. Rare, severe scoliosis with a Cobb angle over 40 degrees with respiratory difficulties leaves open surgery as the only option. However, surgical treatments often suffer from various complications. This case report presents an elderly woman with severe scoliosis at a Cobb angle of 172°. The patient showed no respiratory difficulties. As the patient refused to receive surgical treatment, conservative care was performed. A series of treatments showed positive outcomes to improve the quality of the patient’s life. Extremely severe scoliosis with a 172° Cobb angle has never been reported in the geriatric population. Our case supports the efficiency of conservative management for such severe scoliosis.

## Introduction

As per 2016 guidelines for the management of idiopathic scoliosis by the International Society on Scoliosis Orthopaedic and Rehabilitation Treatment, the goal of conservative treatment is to correct and maintain curve growth during puberty. Bracing is advised for patients with growth curves greater than 20°±5° Cobb angle and should be worn until the spinal bone development has reached maturity [1]. Bracing is not effective for preventing curve progression in skeletally mature patients but may be worn to provide some pain relief. A Cobb angle greater than 50° is associated with an increased rate of further deterioration of the spinal structure and tissue degeneration throughout adulthood, thus largely relying on surgical intervention [2].

## Case Report

**Figure 1.**
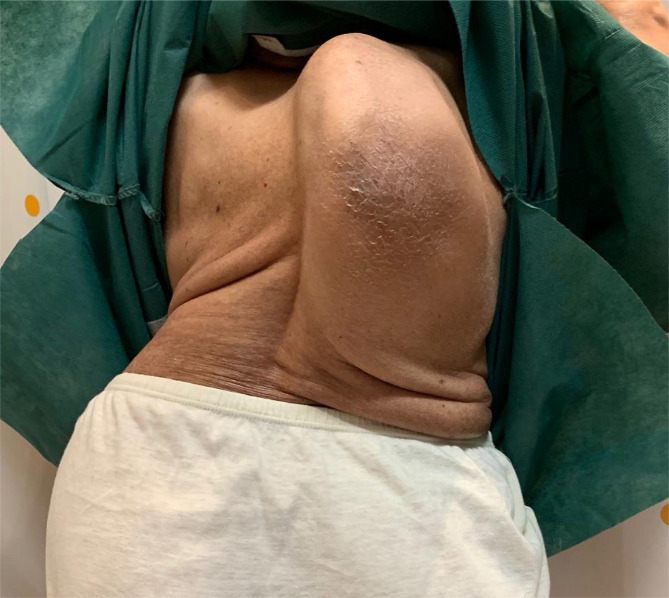
Photograph of the outer appearance of the patient, calcification of skin appeared at the right posterior rib cage area.

A 67-year-old woman sought conservative management for intermittent bilateral lower back pain and numbness of lower limbs, not responding to non-steroidal anti-inflammatory drugs (NSAIDs) and Schroth scoliosis exercises. Although she suffered from difficulties in locomotion, she could walk without falling. The dull back pain rated 6/10 on a 0–10 Numeric Pain Scale (NPS) that could be temporarily relieved with a massage. The patient described the paresthesia and leg pain at a 6/10 scale on NPS, mainly on the right side, accompanied by muscle tightness, compromised balance, which was exacerbated in the sitting position. Considering the patient’s medical history, she was diagnosed with retinoblastoma at 4 years old, and the surgical and chemotherapeutic interventions for the disease resulted in bilateral vision loss. The patient also developed a curvature of her spine in her adolescence. The patient has no history of tuberculosis or polio. She is taking medications for hypertension and calcium/potassium supplements for osteopenia. There is no abnormal sign from her yearly pulmonary and cardiology examinations. A patient’s family member described that her scoliosis and posture had significantly worsened in the past 5 years. The patient shows no interest in her appearance of spinal deformity in consideration of her age and blindness. The patient showed a waddling gait and hunchback posture with an anterolateral head deviation. She had to be supported with a cane and visual assistance from her sister. She was short-statured with distortion of her trunk and a head tilt towards the right (Figure 1). Her breastbone was shifted anteriorly, and the posterior rotation of her right scapula with a positive Adam’s test. Muscular palpation revealed tautness of the right rhomboid, right levator scapulae, quadratus lumborum, and paraspinal muscles. Spinal palpation showed intersegmental restriction of all thoracic segments. Her sensory examination and deep tendon reflexes are intact, but muscle strength was rated 4 out of 5, and the lumbar range of motion was limited at 10° in extension (normal=20–35°) and 20° in flexion (normal=40–60°). She also measured her quality of life (QoL) using the WHOQOL questionnaire scale (Table 1). Despite her conditions, the patient was mentally proactive and well-articulated.

**Table 1.** Significant improvement of the patient’s physical, psychological, social relationship, and environmental health evaluated by the WHO Quality of Life Questionnaire Scale on the first consultation, 6^th^ month, and 24^th^ month evaluation.

**WHOQOL**	**1^st^ Evaluation**	**6^th^ month**	**24^th^ month**
**Physical health**	28/100	68/100	70/100
**Psychological**	54/100	94/100	96/100
**Social Relationships**	49/100	68/100	62/100
**Environment**	56/100	68/100	70/100

**Figure 2.**
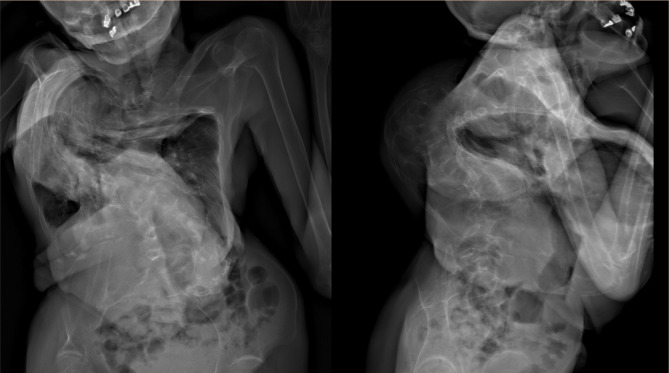
Anteroposterior (left) and lateral (right) full-spine radiographs with a major thoracic curve in a 67-year-old woman. Note the severity of the curve in the coronal plane at Cobb angle 172° and the sagittal plane at 150°.

A weight-bearing, full spine radiograph (EOS image) showed kyphoscoliosis with a right-sided head tilt and spinal convexity, crowding of left-sided ribs, and sclerotic changes on the bilateral tibia, in addition to bilateral genu valgum. The thoracic spine was curved in the lateral plane at 172° Cobb angle (Figure 2), with the convexity directed towards the right. The upper portion of the thoracic spine was bent to the extreme right edge of the thoracic cavity. This created a rotatory effect on the spine, pulling the left side of the body and its organs upwards, consequently leading to the left-sided rib crowding. The anterolateral deviation of the head and neck acted as a counterbalance to this unusual distortion of the thoracic and abdominal cavities. Magnetic resonance imaging (MRI) of the spine corroborated with radiographic findings and showed significant superior displacement of the left kidney. Routine blood investigations and the DEXA scan turned out to be normal, suggesting no other pathology.

Keeping in view the patient’s history of retinoblastoma, absence of any muscular disorder/dystrophy or congenital asymmetry of vertebrae, and other genetic tissue disorders, the patient was diagnosed as an extremely severe case of pre-existing adolescent idiopathic scoliosis (AIS).

The patient was firstly recommended an option of surgical fusion that was refused. She also refused any treatment with aesthetic management of deformity and curvature corrections. Subsequently, the patient was managed conservatively to increase mobility, reduce neurological symptoms, and prevent further degeneration. The patient received a combination of motorized intersegmental decompression, motorized flexion-distraction therapy in thoracic rotation, interferential therapy, instrument-assisted soft tissue mobilization, and gentle manual mobilization. In addition, strengthening and stretching exercises for the stability of the spine involving the paraspinal muscles, rhomboids, trapezius, quadratus lumborum, and gluteal muscles were prescribed for 6 months to reduce the tightness and hypertonicity.

After 32 treatments over the six months, the patient reported a significant pain reduction. A complete resolution of numbness was reported by the third week. At the end of the treatment cycle, the patient could walk without a cane and reported remission of all neurological deficits, with only mild myalgia as a remaining symptom. The hunchback posture appeared to be stable, and the patient was able to maintain balance and walk unaided. Although the right-sided anterolateral deviation of the neck also showed a notable improvement, the deviation showed incomplete resolution. Her WHOQOL improved, and all spinal range of motion recovered to normal. She was advised to continue a scoliosis-home exercise program with monthly maintenance treatments to prevent future deterioration. At the 24^th^ monthly re-evaluation, the patient still had a high QoL, and the outer appearance of spinal deformity remained constant.

## Discussion

Most adult scoliosis continues from an adolescent scoliosis case. After complete skeletal growth, progression of scoliosis can occur as the result of degenerative scoliosis, progressive anomalies, or secondary to other comorbid conditions. Retinoblastoma is a rare malignant tumor that can occur during childhood. The retinoblastoma gene (RB) mutations are observed in almost all retinoblastomas and are strongly linked to osteosarcomas and small cell lung carcinomas. The gene product, retinoblastoma protein (pRb), plays a key role in regulating osteoblast differentiation and has been shown to possess significant tumor-suppressive properties [3]. Berman *et al.* observed that a mutation in the RB gene causes the abnormal development and impaired ossification of several bones, correlating with impairment in osteoblast differentiation [3]. Regarding this case, the patient had bilateral retinoblastoma as a child and developed progressive scoliosis since her adolescence. It is assumed that this retinoblastoma survivor has the risk of genetic predisposition to spinal deformity.

Severe scoliosis is a debilitating condition that has a substantial impact on the quality of life (QoL) of patients. QoL is described by the WHO as “an individual’s view of their place in life in relation to their objectives, aspirations, standards, and concerns in the context of the culture and value systems in which they live” [4]. Most patients have no symptoms when they are diagnosed. Back pain is the result of spondylitis, weakness of the core musculature, and nerve irritation. However, unaware and untreated scoliosis deteriorates as people age and frequently affects the QoL of patients in their later life. In addition, scoliosis patients receiving conservative treatments may feel social isolation, sadness, and reduced leisure activity participation [5]. As a result, scoliosis is widely accepted as a significant risk factor for psychological distress and poor QoL. Therefore, assessing QoL in patients with scoliosis has become a crucial part of evaluation, asides from curvature progression and aesthetic value [6]. As QoL is largely associated with a patient’s ability to enjoy life and achieve personal development and individual goals, the primary goals of scoliosis treatment are set to slow the progression of abnormalities in consideration of QoL with individual relevance [7]. Although the decrease of spine curvature is unlikely to occur in severe adult scoliosis, conservative therapy at this age has its advantages in reducing pain and neuropathy and improving QoL and physical functions. Conservative treatment options for scoliosis range from bracing and exercise to massage therapy and spinal manipulation therapy [8]. Spinal manipulation therapy is efficient in treating musculoskeletal complaints by releasing the entrapped synovial fold within the joint, reducing nerve root encroachment, suppressing inflammatory mediators, and increasing beta-endorphin production [8]. The treatments mentioned above have certain effects on addressing pain and associated anxiety [8]. As demonstrated in this patient with tautness of the paraspinal muscles induced by biomechanical factors of spinal deformity, the conservative treatment appeared to effectively reduce stress, increase core strength and mobility, and achieve overall QoL.

Scoliosis deterioration may be prevented through regular rehabilitation programs. However, there are limited reports on the conservative management for adult patients who refuse to receive surgical procedures despite severe scoliosis throughout their lives. Conservative management, including scoliosis exercises, massages, and spinal traction, can effectively reduce the Cobb angles and slow down the progression of the curvature. For moderate adolescent scoliosis, the average improvement in Cobb angle is 21.2% [9]. However, manual therapies should be utilized with ample caution in geriatric patients with severe scoliosis with possible osteoporosis. Ruling out other pathologies is of utmost importance before conservative management in patients with severe scoliosis.

## Conclusion

In conclusion, this report describes a case where conservative management was satisfactory in a patient with severe (AIS). AIS over 100° is rarely survived 50 years of age. The main limitation of this report was the lack of references for comparative analysis. Further studies are needed to investigate the efficacy of conservative management in severe AIS. Improving QoL as the main indication is recommended to guide the decision-making of severe AIS patients who refuse surgery.

## Acknowledgements

### Conflict of interest

The author declares no conflict of interest.

#### Consent to publish

Written informed consent was obtained from the patient’s guardian to publish this case report and any accompanying images.

#### Data availability

Further data is available from the corresponding author on reasonable request.

#### Authorship

EC contributed to the case information, drafted the manuscript, and prepared the figures. The author has read and approved the final manuscript.
